# The Effect of Negative Feedback on Positive Beliefs in Self-Deception

**DOI:** 10.3389/fpsyg.2019.00702

**Published:** 2019-04-05

**Authors:** Juan Liu, Wenjie Zhang, Youlong Zhan, Lixin Song, Peipei Guan, Dan Kang, Jie Jian, Ronghua Cai, Mei Li

**Affiliations:** ^1^Cognition and Human Behavior Key Laboratory of Hunan Province, Hunan Normal University, Changsha, China; ^2^School of Education Science, Hunan Normal University, Changsha, China; ^3^Department of Psychology, Hunan University of Science and Technology, Xiangtan, China; ^4^School of Design, Hunan University, Changsha, China; ^5^Department of Psychology, McGill University, Montreal, QC, Canada

**Keywords:** self-deception, positive beliefs, forward-looking paradigm, negative feedback, cheating

## Abstract

In the present study, we applied the forward-looking paradigm to examine how positive beliefs appear in self-deception and to further reveal the influence of negative feedback on positive beliefs to decrease self-deception. In Experiment 1, the answer group (with answer hints provided below the test material) and the control group (without answer hints) completed two tests. Participants estimated their Test 1 scores, predicted their performance on the upcoming Test 2 without answer hints, and completed Test 2. Their actual scores on the two tests were recorded. The results showed that the answer group predicted higher Test 2 scores than the control group, but the two groups did not differ in their actual scores. These results showed that the answer group had positive self-deception. In Experiment 2, the two groups were given negative feedback (vs. no feedback) after Test 1, and the changes between their estimated scores on Test 1 and their predicted score and actual score on Test 2 were measured. The results indicated that there was no significant difference in the estimated scores and the predicted score between the two groups under the feedback condition compared with the negative feedback condition. These findings demonstrated that the effectiveness of the forward-looking paradigm can activate participants’ positive beliefs and cheat behaviors by providing the answers to induce self-deception, and negative feedback can decrease the occurrence of self-deception by reducing the positive beliefs of individuals and improving self-awareness to prevent or eliminate the negative impact of self-deception.

## Introduction

Self-deception is considered a positive belief about the self that persists despite specific evidence to the contrary (Mitchell, 2000; Mele, 2002). Many studies about biased self-evaluation suggest that people are motivated to overestimate their abilities or to believe that they are doing better than they truly are (Burson et al., 2006). This motivation is so strong that individuals rationalize or ignore negative evaluations of themselves to uphold a positive belief (Norton, 2009). For example, overconfidence that overestimates one’s actual abilities is a form of self-deception and positive belief (Li et al., 2016). In our view, no overconfidence process is fully self-deceptive. Self-deception is a special case in which an individual maintains a positive self-view and evaluation when faced with negative information. Some researchers believe that self-deception is an act of focusing on the positive to defend oneself and repress the influence of negative feedback to adjust one’s mental state (Fan et al., 2017), to fortify self-enhancement and self-confidence, and to recalibrate an individual’s imbalanced cognition to improve the fit with his or her own perception (Lopez and Fuxjager, 2012; Seiffert-Brockmann and Thummes, 2017).

As of now, knowledge on self-deception mainly comes from two sources: laboratory researches and assessment of scales. Earlier studies mainly obtained data from the self-deception (SD) and impression management (IM) scales from the Balanced Inventory of Desirable Responding (BIDR, Paulhus, 1984). These two scales are widely used as credible assessments of individual self-deception and impression management. Moreover, methods for studying self-deception mainly include retrospective paradigms and forward-looking paradigms. The retrospective paradigms generally focus on measuring the inconsistency between an individual’s evaluation of past experience and the actual behavior of a real event. For example, Quattrone and Tversky (1984) used a classical pain experiment that asked participants to report whether their self-evaluated pain tolerance was affected by the relationship of pain tolerance and heart disease that was previously described to them. The scores of the pain experiment showed that all participants denied the existence of this influence, but their actual behavior proved that they were affected. Participants who were told that a higher pain tolerance means a healthier heart indicated much higher levels of self-evaluated pain tolerance than other participants who were not told so (Quattrone and Tversky, 1984). Gur and Sackeim (1979) adapted a voice recognition task to examine self-deception. Participants were asked to distinguish their own voice from many other voices that had been given positive or negative evaluations, and then they reported “mine” or “not me” for each voice while being connected to a polygraph to test their emotional reaction. This experiment found that participants tended to recognize voices with positive evaluations as themselves and negative evaluations as others, but their physiological response measured by the polygraph was inconsistent with the subjective judgment. Lie detector monitoring showed that the participants’ physical reactions to their own voices or other participants’ voices differed from their subjective reports; that is, the physiological indicators measured by the polygraph showed that the subjects’ physiological responses to the sounds were inconsistent with their subjective reports (Gur and Sackeim, 1979). Additionally, Sloman et al. (2010) used the dot-tracking task to explore self-deception. The dot-tracking task was a video game in which participants started with the cursor on the left side of a computer screen and moved the cursor as fast as possible to a dot that appeared in a random position on the right half of the screen. After completing the initial phase, the fast group was given the instruction that people who moved the cursor faster tended to have higher than average general IQ (intellegence quotient) scores. The slow group was given the instruction that people who moved the cursor slower tended to have higher than average general IQ scores. Then, the participants completed the task in the test phase when the computer screen displayed vague speed feedback (fast, slow, or normal) according to the participants’ speed of cursor movement. The results showed that there was no difference in cursor speed between the two groups in the initial phase. Under the influence of the instructions, the cursor speed of the fast group was significantly higher than that of the slow group. However, all the participants denied that they were affected by the instructions. Accurate speed feedback was provided to the participants in the second experiment. The results showed that there was no significant difference between the fast group and the slow group in the test phase in the second experiment. That is, accurate feedback reduced the occurrence of self-deception. This dot-tracking paradigm established the theory that self-deception occurs under ambiguous conditions, as has been widely recognized. Although the above experimental methods illustrate the classic paradigm for studying self-deception, they cannot be used to measure the unconscious processes of self-deception (Ren et al., 2018) or applied stably and repeatedly to follow-up studies of self-deception. Therefore, advances in the experimental paradigms are an important prerequisite for further exploration of self-deception.

Recently, Chance et al. (2011) applied the forward-looking paradigm to investigate the mechanism of self-deception. In this paradigm, participants were informed of the correct answer before they answered the questions, which offered them the chance to cheat and obtain a better score. Researchers regard this process of seeing answers as cheating (Chance et al., 2011, 2015). The participants overestimated their future test scores by self-deception. These participants chose to deceive themselves into believing that better scores were obtained not because they had the answers beforehand but because of their actual talent. This experiment consisted of two knowledge tests. Participants completed 10 knowledge questions in Test 1 (e.g., “How many US states border Mexico?” and “In which US state is Mount Rushmore located?”), estimated the scores of Test 1 and predicted their future scores on Test 2 (100 knowledge questions similar to Test 1). Specifically, in Test 1, the answer group had the opportunity to see the answer at the bottom of the question sheet, while the control group did not. Then, both groups of participants continued to Test 2, for which neither group had answer keys. Through a comparison of the difference between the actual and estimated scores on Test 1, the behavioral aspects of cheating and establishing positive beliefs could be examined. If both the actual scores and the estimated scores of the answer group were significantly higher than those of the control group, then it could be deduced that the answer group was cheating and established a positive belief: “I am good at this task.” Self-deception was examined through the difference between the predicted scores and the actual scores for Test 2. If the scores predicted by the answer group were significantly higher than those predicted by the control group and the actual scores were not different, this result indicated that self-deception occurred in the answer group. The results showed that the actual scores and the estimated scores of the answer group were significantly higher than those of the control group, which indicated that the answer group was cheating and established a positive belief in Test 1. In Test 2, the prediction scores made by the answer group were significantly higher than those by the control group, and the true scores were not significantly different. The answer group deceived themselves into believing that they would perform better in the future than they actually did (holding positive beliefs about their ability being better than their actual ability) despite knowledge of negative evidence, such as “I saw the answer.”

Deceiving others is beneficial, but why do people deceive themselves? Evolutionary psychologists believe that self-deception can help individuals ignore clues such as cognitive load, conscious repression, and tension in order to better deceive others (von Hippel and Trivers, 2011). Deception and self-deception are like two sides of the same coin that are mutually dependent and interactive. Self-deception slowly becomes a strategy to persuade or deceive others without being detected, and then deceiving others also becomes a means of self-deception (Dings, 2017). In the forward-looking paradigm, participants do not interpret their cheating behavior as “I am a liar,” a negative belief, but use the positive scores of deception to enhance their positive beliefs (“I am capable”) and the logical belief that “I am capable, I am not a cheater.” The forward-looking paradigm could not only be used to study self-deception in individuals but also be extended to interpersonal self-deception. Such an approach would be easy to carry out and could avoid the difficult problem of the retrospective paradigm, which is not having an objective measure of unconscious decisions. Chance et al.’s (2011) experiment is based on knowledge of material familiar to people in the United States, but it is not applicable for Chinese participants more broadly. Furthermore, some studies have shown that there are significant differences in social cognition and behavior between people in the East and the people in the United States (Norenzayan and Nisbett, 2000; Yukiko and Shinobu, 2009). Hence, not only does the forward-looking paradigm experimental material need to be studied, but the validity of the experimental paradigm must also confirm in the Eastern cultural context. Therefore, the first purpose of this study was to verify the effectiveness of the forward-looking paradigm to induce self-deception in the context of Chinese culture and to expand and enrich the experimental material.

In addition, many studies have proven that self-deception has positive effects on individuals, such as improving subjective well-being (Ford, 2004; Lopez and Fuxjager, 2012) and increasing self-confidence (Ren et al., 2018) and self-perceived personal charm (Linton and Wiener, 2001). However, other studies have shown that self-deception has a negative effect on individuals. The negative impact of self-deception on individuals makes it impossible for individuals to clearly recognize themselves, and self-deception is not conducive to long-term development. Self-deception has benefits from a short-term perspective, but there is a high price to pay in the long term (Chance et al., 2011; Lauria et al., 2016). Self-deception can be misleading for social policy and may cause disasters for groups and society; war is the most expensive price that we have paid, as illustrated by Hitler’s Nazi party (Trivers, 2000). Self-deception can be a strategy of moral hypocrisy to misperceive one’s behavior as moral and avoid comparing one’s behavior with moral standards (Batson et al., 1999). Self-deception promotes unethical behavior, cheating, the bankruptcy of enterprises and governments (Chance et al., 2015), corrupt behavior (Desai et al., 2017), and the undermining of corporations (Babino et al., 2018). Thus, it is important to prevent and eliminate the negative impact of self-deception. It is of great theoretical and practical significance to explore how reducing such costly self-deception helps individual better monitor their self-deception behavior to prevent individual losses and prevent the harmful effects of self-deception on society.

How can self-deception be decreased? Self-deception is considered a positive belief about the self that persists despite specific evidence to the contrary. In our view, self-deception can be decreased by weakening the positive beliefs of self-deceivers. How do these positive beliefs change? According to belief adjustment theory, when people find new information that conflict with their original beliefs, they will adjust their original beliefs to accept the inconsistent information to adapt to the new environment. When confronted with inconsistent information, individuals readjust the strength of their belief rather than completely revising their original belief (Johnson-Laird et al., 2004). Does positive belief in self-deception follow belief adjustment theory? That is, will this positive belief be weakened when people face inconsistent negative beliefs? We examine the decline of self-deception by manipulating the negative feedback to influence positive beliefs in self-deception. We support the theory suggested by Sloman (2011) that self-deception depends on ambiguous conditions. However, it remains unclear how negative feedback affects self-deception in the forward-looking paradigm, and non-feedback has the greatest ambiguity with regard to self-deception. Therefore, the second purpose of this study was to provide relatively truthful and accurate feedback to the participants and to examine whether the answer group would be aware of the good scores obtained by cheating on Test 1 and whether their predictions for Test 2 would be closer to the control group’s scores. According to the theory of belief adjustment and the idea that self-deception depends on ambiguous conditions, we hypothesize that self-deception can be decreased by weakening individuals’ positive beliefs.

Based on the above, this study used a forward-looking paradigm to examine how the positive beliefs of self-deception occurs and to further explore the impact of negative feedback on that positive beliefs to decrease self-deception.

## Experiment 1: Positive Beliefs in Self-Deception in the Forward-Looking Paradigm

### Purpose and Hypothesis

The purpose of this study was to examine how positive beliefs occur in the forward-looking paradigm during self-deception. The study assumed that compared to the control group participants, the participants in the answer group would establish positive beliefs about their scores because of cheating in Test 1 and predict higher scores for their future Test 2 to show self-deception.

### Methods

#### Participants

The experimental procedure was approved by the IRB of the Institute of Psychology, Hunan Normal University. A total of 47 college students (19 male and 28 female, average age 22.48 ± 0.69 years) were recruited, none were psychology majors. All participants were right-handed, with normal or corrected-to-normal vision. They were randomly divided into the answer group (25 people, with answer hints) and the control group (22 people, without answer hints). All participants signed their written informed consent to the experiment and were given appropriate compensation after the experiment.

#### Experimental Design

A 2 (group type: answer group vs. control group) × 2 (score: estimation/prediction scores vs. actual scores) mixed design was conducted in Experiment 1. The group type was the between-subject variables, and the within-subject variable was the score. The dependent variable was the estimated and actual scores for Test 1 and the predicted and actual scores for Test 2.

#### Material

**Figure 1:**
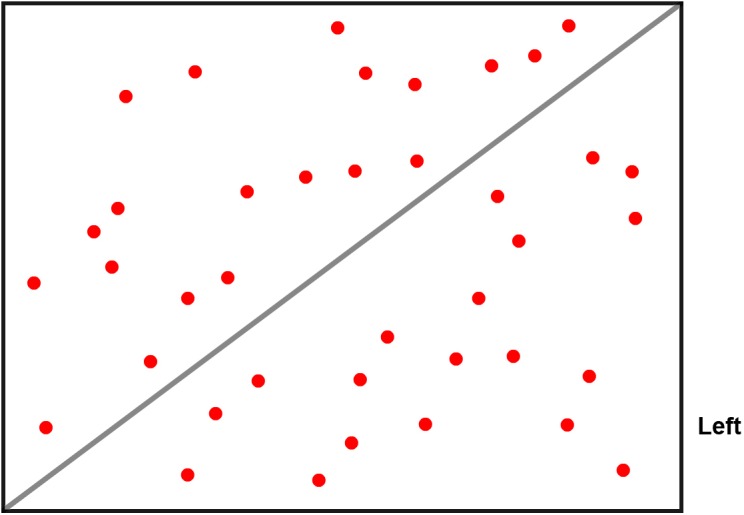
A dot graph with an answer hint.

Dot estimation task: Each graph is a problem, and there are 60 rectangular red dot graphs (see Figure 1 for an example). Ten of the 60 graphs had an answer hint written at the bottom right corner and were used for Test 1 by the answer group, and the same type of graphs without answer hints was used for Test 1 by the control group. The remaining 50 graphs without the answer hint were used for Test 2 for both groups. Each graph is a rectangle divided into two halves by a diagonal line. In each graph, 39 or 40 red dots are evenly distributed on both sides of the diagonal line. To increase the ambiguity of the answer, the number of points on both sides of the graph was either nearly equal or equal. There were three types of answers: more dots the left side, more dots on the right side and an equal number of dots on each side. Among these graphs, 20 graphs have the same number of dots on both sides, 20 graphs have one more dot on the right side, and the remaining 20 graphs have one more dot on the left side. The task of estimating the number of points without counting is quite difficult, and the answer is chosen based on intuition. The red dots are randomly presented in the graph. Each graph is rendered randomly by a computer program. Each graph appears as a trial, and the time taken for each trial is 6 s. The participants would press the “F” key if they think there are more dots on the left side, “J” for the right side, and “Y” for an equal number of dots.

#### Procedure

**Figure 2:**
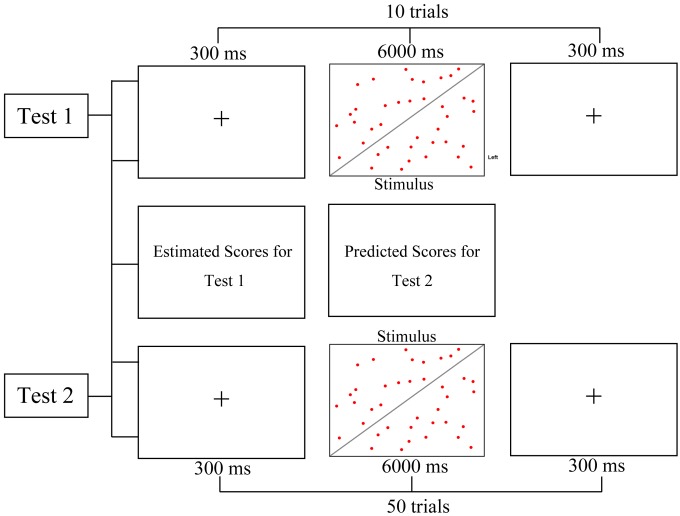
Procedure of Experiment 1.

Forty-seven participants completed the test on computers equipped with Eprime 2.0. The test consisted of Test 1 and Test 2 (see Figure 2). Before the test, a sample dot estimation task with instructions was presented to participants, and the participants were informed about the requirements and specific operations of the test. Participants were informed that the dot estimation task was designed to investigate the visual observation ability of the college students. In the answer group, the participants were also given this instruction “There is an answer below and to the right of the screen; you can check your answer, but please do your own work.” This was an ambiguous instruction, they did not forbidden to look at the answer, but they did imply that using the answer hint to answer the question would be wrong (Chance et al., 2015). The control group completed the same test task but without the answer hints and the ambiguous instructions. After the participants completed Test 1 with 10 questions, they needed to estimate their score for Test 1 and predict their future score for Test 2, which had 50 graphs, by entering the scores on the computer screen. Finally, they completed Test 2 with no answer hints. In Test 1, because of the influence of the answer hint (participants of the answer group saw the answer hint, the control group did not have the answer hint), the answer group had a significantly higher score than the control group on both the estimated scores and actual scores. If the actual score of the answer group was significantly higher than that of the control group, this result indicated that the answer group was cheating by looking at the answer; if the estimation of the score made by the answer group was significantly higher than that of the control group, this result meant that the answer group had set up a positive belief: I am good at this test. In Test 2, these participants chose to deceive themselves into believing that the better scores were obtained not because they had the answers beforehand but because of their actual talent. Then, participants of answer group more estimated their scores in Test 2 by self-deception than those of the control group. If the predicted scores made by the answer group were significantly higher than those of the control group but the actual scores were not different, then the answer group was self-deceptive; that is, they had a positive belief and still persisted in this belief in the face of the opposite evidence.

### Results

#### Estimated and Actual Scores for Test 1

**Figure 3:**
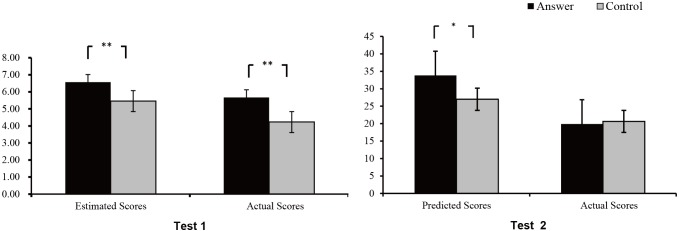
Estimated and actual scores for Test 1 and predicted and actual scores for Test 2 in the control and answer groups. ^∗^*p* < 0.05, ^∗∗^*p* < 0.01, ^∗∗∗^*p* < 0.001.

Repeated-measures analyses of variance (ANOVA) were performed on the estimated scores and actual scores in the two groups for Test 1 (see Table 1 and Figure 3). The results showed that the main effect of the group type was significant, *F*_(1,45)_ = 14.33, *p* < 0.001, *η*_p_^2^ = 0.24, and the scores of the answer group were higher than those of the control group. There was a significant main effect of score, *F*_(1,45)_ = 17.34, *p* < 0.001, *η*_p_^2^ = 0.28, and the estimated scores were higher than the actual scores. There was no significant interaction effect for the group type and the score, *F*_(1,45)_ = 0.33, *p* > 0.05, *η*_p_^2^ = 0.01.

**Table 1:** Estimated and actual scores for test 1 and predicted and actual scores for test 2 (*M* ± *SD*).

	Test 1		Test 2	
	Answer	Control	Answer	Control
Score	6.57 ± 1.50	5.46 ± 1.42	33.81 ± 9.47	27.00 ± 9.62
Actual Score	5.67 ± 2.15	4.23 ± 1.17	19.90 ± 2.37	20.65 ± 3.81

To further verify whether the answer hints could cause cheating behavior and positive beliefs, we used an independent-sample *t*-test to compute the difference of the estimated scores and the actual scores between the two groups. The results showed that the estimated scores of the answer group were higher than those of the control group, *t*(45) = 2.84, *p* = 0.007, *conhen’s d* = 0.76, and the actual scores of the answer group were significantly higher than those of the control group, *t*(45) = 3.07, *p* = 0.005, *conhen’s d* = 0.71.

#### Predicted and Actual Scores for Test 2

Repeated-measures ANOVAs were performed on the predicted and actual scores in the two groups for Test 2 (see Table 1 and Figure 3). The results showed that the main effect of the group type was significant, *F*_(1,45)_ = 5.26, *p* < 0.05, *η*_p_^2^ = 0.11, and the scores of the answer group were higher than those of the control group. There was a significant main effect of the score, *F*_(1,45)_ = 52.52, *p* < 0.01, η_p_^2^ = 0.54, and the predicted scores were higher than the actual scores. There was a significant interaction effect for the group type and the score, *F*_(1,45)_ = 7.32, *p* < 0.01, *η*_p_^2^ = 0.14. We conducted a simple effect analysis for the answer group and the control group. The results showed that the answer group had higher predicted scores than the control group, *F*_(1,45)_ = 7.11, *p* < 0.05, and the actual scores of the two groups were not significantly different, *F*_(1,45)_ = 0.64, *p* > 0.05. In addition, the predicted scores of the answer group and the control group were significantly higher than the actual scores, *F*_(1,45)_ = 44.76, *p* < 0.001, *F*_(1,45)_ = 11.54, *p* < 0.001.

To further verify whether the answer hints could affect the actual scores of the answer group to induce self-deception by the participants’ cheating, we converted raw scores to percentages and used a paired-sample *t*-test to compute the difference between the scores and the actual scores in Test 1 and Test 2 for the two groups. The results showed that the percentages of estimated scores for Test 1 were not significantly higher than the percentages of predicted scores for Test 2 in the answer group, *t*(24) = -0.66, *p* = 0.52, *conhen’s d* = -0.15, and the percentages of actual scores for Test 1 were significantly higher than the percentages of predicted scores for Test 2, *t*(24) = 3.99, *p* = 0.001, *conhen’s d* = 1.24. In contrast, the percentages of estimated scores for Test 1 were not significantly higher than the percentages of predicted scores for Test 2 in the control group, *t*(21) = 0.22, *p* = 0.83, *conhen’s d* = 0.06, and the percentages of the actual scores for two tests were not significantly different, *t*(21) = 0.50, *p* = 0.62, *conhen’s d* = 0.11.

### Discussion

In Experiment 1, the main effect of the group type was significant, and the actual scores of the answer group were significantly higher than those of the control group in Test 1, which indicated that the answer group was affected by the answer hints and cheated by seeing the answers. The answer group had higher estimated scores than the control group in Test 1, which indicated that the participants in the answer group had established positive beliefs. In Test 2, the main effect of the group type was significant, the predicted scores of the answer group were significantly higher than those of the control group, and the actual scores were did not differ between the groups, which indicated that the answer group occurred self-deception. These results are not only consistent with the previous hypothesis but also consistent with the study by Chance et al. (2011). By ignoring the influence of negative evidence, people deceive themselves to maintain a positive belief about their future performance to reflect their abilities (Chance et al., 2011, 2015).

In addition, the main effect of score was significant in the two tests, and the estimated or predicted scores were higher than the actual scores. These findings indicate that the participants in the two groups overestimated their actual ability and that they enhanced and maintained a positive self-concept by evaluating their traits above their actual level (Greenwald, 1980; Taylor and Brown, 1988; Burson et al., 2006). However, there was no significant difference in the percentages of scores between Test 1 and Test 2 in the control group and a significant difference in the percentages of actual scores between Test 1 and Test 2 in the answer group. These results support the effect of the answer hints on the answer group. That is, the positive beliefs of the control group were not self-deception, but the positive beliefs of the answer group when they were faced with the negative evidence (answer hints) were regarded as self-deception. However, the question remains whether this positive belief can be changed by presenting opposing negative beliefs. Therefore, Experiment 2 further explored the variability of positive beliefs in self-deception by investigating the effect of negative feedback on positive beliefs in self-deception. In Experiment 1, the average actual scores for Test 2 in the two groups were 20.65 (the control group) and 19.9 (the answer group). Both groups had scores lower than their self-evaluation, so the feedback in Experiment 2 was the negative feedback of the “low test scores of your test”.

## Experiment 2: the Effect of Negative Feedback on Positive Beliefs in Self-Deception

### Purpose and Hypothesis

The purpose of this experiment was to examine the effect of negative feedback on positive belief in self-deception. We hypothesized as follows: (1) compared to the control group participants, the participants in the answer group would establish positive beliefs about their higher scores because of cheating in Test 1 and predict higher scores for their future Test 2 to show self-deception under the no-feedback condition; (2) compared to the control group participants, the participants in the answer group would reduce positive beliefs about their scores in Test 1 to decrease the occurrence of self-deception in Test 2 under the negative-feedback condition.

### Methods

#### Participants

The experimental procedure was approved by the IRB of the Institute of Psychology, Hunan Normal University. A total of 93 college students (40 male and 53 female; average age 23.45 ± 0.71 years) were recruited. The participants were all right-handed, had normal or corrected-to-normal vision, and were randomly assigned to the answer group (28 participants in the negative feedback condition, 20 participants in the no-feedback condition, with answer hints) and the control group (25 participants in the negative feedback condition, 20 participants in the no-feedback condition, without answer hints). All participants signed their written informed consent for the experiment and were given appropriate compensation after the experiment.

#### Experimental Design

A 2 (group type: answer group vs. control group) × 2 (feedback condition: negative vs. no-feedback) × 2 (score: estimation/prediction scores vs. actual scores) mixed design was conducted in Experiment 2. The group type and feedback condition were the between-subject variables, and the within-subject variable was the score. The dependent variable was the estimated and actual scores for Test 1 and the predicted and actual scores for Test 2.

#### Material

The same dot estimation task material used in Experiment 1 was used for Experiment 2.

#### Procedure

**Figure 4:**
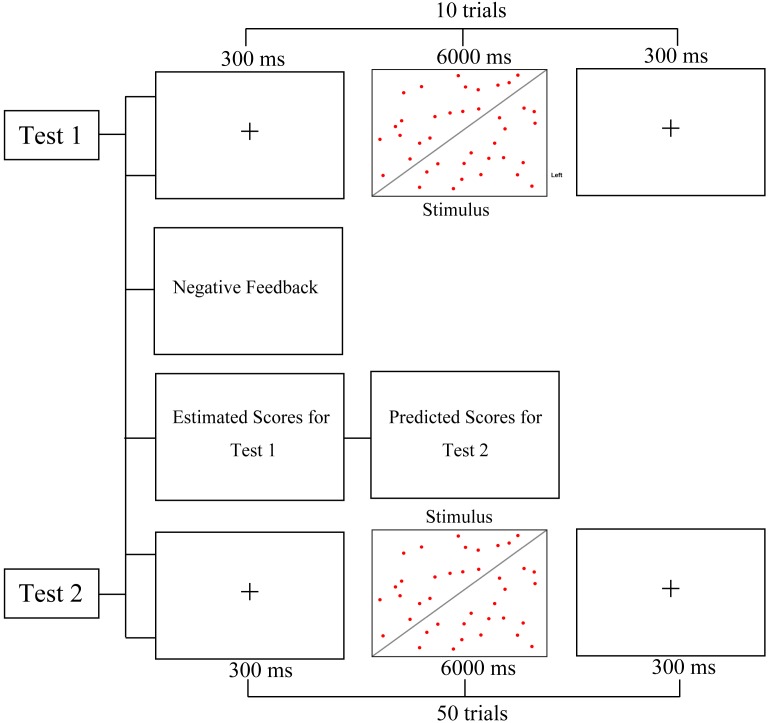
Procedure of Experiment 2.

At the end of Test 1, the computer gave the participants negative feedback that said “low test score for your test.” Then, the participants were asked to estimate their scores for Test 1 and predict their scores for Test 2. The other procedures were the same as those in Experiment 1 (Figure 4).

### Results

#### Estimated and Actual Scores for Test 1

Repeated-measures ANOVAs were performed on the feedback condition and score in the two groups for Test 1 (Table 2). The results showed that the main effect of the group type was significant, *F*_(1,89)_ = 12.76, *p* < 0.001, *η*_p_^2^ = 0.13, and the scores of the answer group were higher than those of the control group. There was a significant main effect of the feedback condition, *F*_(1,89)_ = 7.43, *p* < 0.01, *η*_p_^2^ = 0.08, and the score of the no-feedback condition were higher than those of the negative feedback condition. There was no significant main effect of the score, *F*_(1,89)_ = 0.92, *p* > 0.05, *η*_p_^2^ = 0.01, and the estimated scores were not different from the actual scores. There was no significant interaction effect of group type and score, *F*_1,89)_ = 1.99, *p* > 0.05, *η*_p_^2^ = 0.02, and no significant interaction effect for group type, feedback condition and score, *F*_(1,89)_ = 0.93, *p* > 0.05, *η*_p_^2^ = 0.01. However, there was a significant interaction effect for the feedback condition and the score, *F*_(1,89)_ = 15.53, *p* < 0.001, *η*_p_^2^ = 0.15. We conducted a simple effect analysis for the answer group and the control group. The results showed that the estimated scores of the no-feedback condition were significantly higher than those of the negative feedback condition, *F*_(1,89)_ = 25.41, *p* < 0.001, and the actual scores were not significantly different between the two conditions, *F*_(1,89)_ = 0.65, *p* > 0.05. In addition, the estimated scores of the no-feedback condition were higher than the actual scores, *F*_(1,89)_ = 11.98, *p* < 0.01, and the estimated scores of the negative feedback condition were lower than the actual scores, *F*_(1,89)_ = 5.93, *p* < 0.05.

**Table 2:** Comparison of estimated, predicted, and actual scores between groups (*M* ± *SD*).

	Estimated score for Test 1		Predicted score for Test 2	
	Actual score for Test 1		Actual score for Test 2	
	Answer	Control	Answer	Control
Negative	4.57 ± 1.69	4.10 ± 1.37	23.40 ± 6.53	24.90 ± 8.57
Feedback	5.89 ± 2.77	4.25 ± 1.45	19.46 ± 2.73	19.55 ± 4.20
No feedback	6.60 ± 1.50	5.64 ± 0.32	34.00 ± 9.54	27.49 ± 10.13
	5.50 ± 2.35	4.32 ± 1.11	20.05 ± 2.42	20.12 ± 4.52

**Figure 5:**
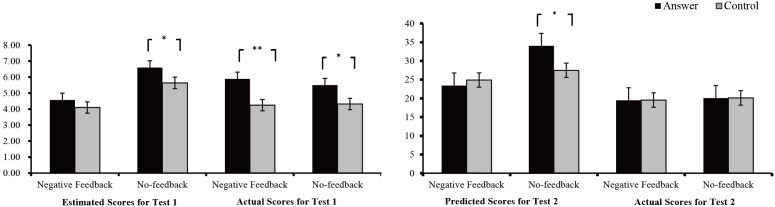
Comparison between the two groups across conditions. ^∗^*p* < 0.05, ^∗∗^*p* < 0.01, ^∗∗∗^*p* < 0.001.

To further verify whether negative feedback could decrease positive beliefs by reducing the estimated scores and whether the answer hints could cause cheating behavior and positive beliefs, we used an independent-sample *t*-test to compute the difference between the estimated scores and the actual scores between the two groups (see Figure 5). The results of the negative feedback condition showed that the estimated scores of the answer group were not significantly different from those of the control group, *t*(46) = 1.03, *p* = 0.31, *conhen’s d* = 0.31, and the actual scores of the answer group were significantly higher than those of the control group, *t*(46) = 2.67, *p* = 0.01, *conhen’s d* = 0.74. The results of the no-feedback condition showed that the estimated scores and the actual scores of the answer group were significantly higher than those of the control group, *t*(43) = 1.20, *p* = 0.05, *conhen’s d* = 0.89, *t*(43) = 2.07, *p* = 0.04, *conhen’s d* = 0.64.

#### Predicted and Actual Scores for Test 2

Repeated-measures ANOVAs were performed on the feedback condition and score in the two groups for Test 2 (see Table 2 and Figure 5). The results showed that the main effect of the group type was not significant, *F*_(1,89)_ = 1.55, *p* > 0.05, *η*_p_^2^ = 0.02, and the scores of the answer group were not different from those of the control group. There was a significant main effect of the feedback condition, *F*_(1,89)_ = 13.51, *p* < 0.001, *η*_p_^2^ = 0.13, and the scores of the no-feedback condition were higher than those of the negative feedback condition. There was a significant main effect of the score, *F*_(1,89)_ = 58.97, *p* < 0.01, *η*_p_^2^ = 0.40, and the predicted scores were higher than the actual scores. There was a significant interaction effect for group type, feedback condition and score, *F*_(1,89)_ = 4.05, *p* < 0.05, *η*_p_^2^ = 0.04. We conducted a simple effect analysis for the answer group and the control group. The results showed that the predicted scores and the actual scores were not significantly different between the answer group and the control group under the negative feedback condition, *F*_(1,89)_ = 0.92, *p* > 0.05, *F*_(1,__89)_ = 0.03, *p* > 0.05. Under the no-feedback condition, the predicted scores of the answer group were higher than those of the control group, *F*_(1,__89)_ = 4.33, *p* < 0.05, and the actual scores did not differ between the answer group and the control group, *F*_(1,__89)_ = 0.02, *p* > 0.05.

### Discussion

In Experiment 2, the results of Test 1 showed that the main effect of the group type and the feedback condition were significant, and the main effect of the score was not significant, which indicated that the scores of the participants were affected by the negative feedback. Under the no-feedback condition, the estimated scores of the answer group were significantly higher than those for the control group, and the estimated scores were higher than the actual scores, which indicated that the participants cheated on the test by seeing the answer hints and obtained a positive belief. These results are consistent not only with the previous hypothesis and the results of Experiment 1 but also with the study by Chance et al. (2011). Under the negative feedback condition, the estimated scores of the answer group were not significantly different from those of the control group, and the predicted scores were lower than the actual scores. The actual scores of the answer group were significantly higher than those of the control group, which indicated that the negative feedback reduced the positive beliefs in Test 1, even if the participants cheated on the test by seeing the answer hints.

The results of Test 2 showed that the main effect of the feedback condition and score was significant and the main effect of the group type was not significant, which indicated that the positive beliefs of the answer group reduced their scores to the level of the control group because of the impact of negative feedback. There was a difference between Test 1 and Test 2 in the main effect of the score because the actual scores of the answer group reduced by the lack of answer hints provided to the participants in Test 2. Under the no-feedback condition, the predicted scores of the answer group were significantly higher than those of the control group and the actual scores of the two groups did not differ, which indicated that the participants in the answer group occurred self-deception. These results are consistent not only with the previous hypothesis and the results of Experiment 1 but also with the study by Chance et al. (2011). Under the negative feedback condition, the estimated scores and the actual scores of the answer group were not significantly different from those of the control group, which indicated that negative feedback decreased self-deception to the level of the control group. The results of Experiment 2 suggest that negative feedback might have decreased the occurrence of self-deception in Test 2 by reducing the positive beliefs in Test 1 to reduce such costly self-deception.

## General Discussion

The present study used the forward-looking paradigm to examine how positive beliefs appeared in self-deception and further revealed the influence of negative feedback on positive beliefs to decrease self-deception. The findings of Experiment 1 and the no-feedback condition for Experiment 2 showed that the estimated scores and the actual scores of the answer group were higher than those of the control group in Test 1. The predicted scores of the answer group were higher than those of the control group in Test 2, but the actual scores did not differ between the groups in Test 2. However, the findings of the negative feedback condition for Experiment 2 showed that the estimated scores, the predicted scores and the actual scores (Test 2) of the answer group were not significantly different from those of the control group, and the actual scores of the answer group were significantly higher than those of the control group in Test 1. These findings demonstrate that the effectiveness of the forward-looking paradigm can induce self-deception to expand and enrich the experimental material used, and negative feedback may have decreased the occurrence of self-deception in Test 2 by reducing the positive beliefs in Test 1 to prevent or eliminate the negative impact of self-deception.

### The Effectiveness of the Forward-Looking Paradigm to Induce Self-Deception

To further improve and develop the experimental methods to study self-deception, we used computer programs and graph materials to examine the effectiveness of the forward-looking paradigm to induce self-deception and to verify the applicability of the dot estimation material to more general participants. The results of Experiment 1 and the no-feedback condition of Experiment 2 showed that the estimated scores, the actual scores in Test 1 and predicted scores of the answer group were significantly higher than those of the control group, but the actual scores did not differ between the groups in Test 2. These findings indicate that the answer group occurred self-deception. The participants in the answer group deceived themselves into believing that they would perform better in the future than they actually did despite knowledge of negative evidence, such as seeing the answers. These results are consistent with Chance et al.’s (2011) research and prove that the “forward-looking” paradigm can better induce self-deception in an Eastern cultural context. The dot estimation material can induce self-deception and can be widely used in academic settings to study self-deception (Ren et al., 2018). To our knowledge, this is the first time that the forward-looking paradigm has been tested using a computer program and graph materials. Our research results represent an improvement in the experimental methods used to study self-deception. It is expected that improvements in this method can provide an experimental basis for the study of the neural mechanisms of self-deception.

### Negative Feedback Decreases Positive Beliefs in Self-Deception

To effectively prevent the occurrence of self-deception, we provided negative feedback after Test 1 in Experiment 2 to explore whether this negative feedback could decrease self-deception by reducing the positive beliefs of participants. The results of Experiment 2 showed that the estimated scores, the predicted scores and the actual scores in Test 2 of the answer group were not significantly different from those of the control group, and the actual scores of the answer group were significantly higher than those of the control group in Test 1. These results indicated that negative feedback may have decreased the occurrence of self-deception in Test 2 by reducing the positive beliefs in Test 1. Our results are consistent with belief adjustment theory, which suggest that individuals adjust their original beliefs to accept inconsistent information (Johnson-Laird et al., 2004). Most previous studies on self-deception have used the retrospective paradigm to promote the generation of self-deception through negative feedback (Gur and Sackeim, 1979; Quattrone and Tversky, 1984; Sloman et al., 2010). The negative feedback of the retrospective paradigm is a type of threatening information that can induce self-deception in participants by changing their behavior and denying the change of behavior. However, in our research, negative feedback affected the positive beliefs of the participants to reduce the occurrence of self-deception. Therefore, the difference in the results between the retrospective paradigm and our study was due to the difference in the operation of the experimental conditions and the measurement of self-deception. Sloman et al. (2010) found that self-deception occurred under ambiguous conditions, and compared with ambiguous feedback, accurate feedback could effectively reduce the occurrence of self-deception. The findings of our study demonstrated that no-feedback conditions have more highly ambiguous conditions to facilitate the occurrence of self-deception than do relatively accurate negative feedback conditions. Thus, our results also support the theory of Sloman et al. (2010) that self-deception depends on ambiguous conditions. In addition, high self-awareness has been positively correlated with low self-deception (Lynn et al., 2014), and negative feedback might increase the level of awareness of self-competence to reduce positive beliefs in self-deception by improving self-perception.

### Biased Information Processing of Positive Beliefs in Self-Deception

Many studies have shown that individuals tend to overestimate their positive traits of intelligence, capability and morality (Taylor and Brown, 1988). Our study found the same psychological phenomena. The results showed that the main effect of scores was significant in Experiment 1 and Test 2 of Experiment 2, and the predicted scores were higher than the actual scores of the control group and the answer group. These results showed that the participants generally overestimated their real scores due to overconfidence. von Hippel and Trivers (2011) suggested that not all biased processing of information is self-deception, however, when people are consciously inclined toward positive information, unconsciously avoiding negative information and reflecting individual motivation is regarded as self-deception. That is, the positive beliefs of the control group were not self-deception, but the positive beliefs of the answer group when faced with negative evidence (answer hints) were regarded as self-deception. The pursuit of truth is important in human survival and reproduction. Why do individuals have unrealistic positive beliefs about themselves through self-deception? Most researchers now use the theory of biased information processing to explain the mechanism of positive beliefs in self-deception (van der Leer and McKay, 2017). Self-deception can occur at any stage of biased information processing, and people maintain a positive self-evaluation according to their willingness to deviate, ignore their memory and rationalize improper behavior. Furthermore, self-deception can occur at any stage of information processing, and people are biased according to their willingness to extract and block information or reconstruct memories. When individuals search for or receive information, they are sometimes inclined to avoid further searches for information and even automatically question the validity and authenticity of the information because it does not match their own goals and ideas. As individuals attempt to interpret the information they obtain, some unwelcome information may be recoded as being more positive. When information is extracted from memory, it is not guaranteed that unwelcome information is retrieved even if it has been acknowledged or even accepted for encoding. Information that is not consistent with an individual’s own preferences is easily forgotten or misunderstood.

### Logic of the Relationship Between Cheating and Self-Deception

From the interpersonal point of view, some researchers have suggested that self-deception is contributes to interpersonal deception (von Hippel and Trivers, 2011). For example, it has been proposed that self-deception can promote interpersonal persuasion by maintaining positive self-image (Seiffert-Brockmann and Thummes, 2017; Smith et al., 2017), which shows that deception and self-deception are interdependent and mutually promoting. Cheating is a fraudulent way of doing illegal or unregulated things; that is, cheating is a kind of deceit. Therefore, cheating and self-deception are also interdependent and mutually beneficial, and our experiment is in line with this logic. However, people generally think that the problematic behavior of cheating will make individuals feel worse about themselves; that is, if they have the ability to obtain the desired scores, they do not need to cheat. However, in reality, when self-deception appears, the sense of morality will fade (Tenbrunsel and Messick, 2004). Self-deception reduces the cognitive dissonance caused by unethical behavior (Lauria et al., 2016). Self-deception is regard as a strategy to deal with conflict between self-interest and moral standards (Batson et al., 1999; Tang et al., 2017, 2018). Even if unconsciously one thinks of it as a kind of immoral behavior, the conscious mind rationalizes this immoral behavior. People tend to focus on the positive scores of cheating, leading to their neglect of the disagreeable process of cheating in order to maintain positive beliefs for self-view.

### Limitations and Future Study

Previous studies have found that it is difficult for real feedback to reduce self-deception to the level of the control group after repeated feedback (Chance et al., 2015; Tang et al., 2018). However, our research found that one incident of negative feedback decreased self-deception to the level of the control group. This difference might be because the negative feedback in our experiment appeared after Test 1 and before the estimated scores (Chance included feedback after Test 2). Thus, we could reduce the occurrence of self-deception by influencing the establishment of positive beliefs among our participants. Our study did not examine self-deception at different points of feedback (after Test 1 or Test 2), which is one of our limitations. Future research should place negative feedback at different time points in an experiment to test whether self-deception differences exist. Furthermore, the results of our research only explained and examined ubiquitous self-deception with positive beliefs but not broader self-deception, such as negatively twisted self-deception (Mele, 2002; Leeuwen, 2007). In addition, our study did not examine self-deception other than negative feedback types (ambiguous vs. real) and social situations. Future studies can investigate the cognitive mechanism of self-deception depending on ambiguous or real negative-feedback conditions and social situation factors. The neural mechanisms of individual self-deception can be further explored using ERP technology or fMRI technology.

## Conclusion

This study contributes to experimental methods and suggests ways to reduce self-deception. On the one hand, we used computer programs and “dot estimation” materials in the forward-looking paradigm to prove that the effectiveness of the forward-looking paradigm can induce self-deception in an Eastern cultural context, and to expand the experimental material used. On the other hand, our research demonstrated that negative feedback can decrease the occurrence of self-deception by reducing the positive beliefs of individuals and improving self-awareness to avoid or eliminate the negative impact of self-deception on the basis of belief adjustment theory. No-feedback conditions have more ambiguous conditions to facilitate the occurrence of self-deception than do negative feedback conditions. In other words, the forward-looking paradigm is an effective experimental method to explore self-deception by inducing cheating, and negative feedback can decrease self-deception.

## Author Contributions

JL was responsible for the preparation of experimental procedures and wrote the manuscript. YZ and LS analyzed the data. PG, DK, and JJ performed the experimental procedures and organized the participants for the experiments. RC and ML examined the experimental material. WZ reviewed the manuscript.

## Conflict of Interest Statement

The authors declare that the research was conducted in the absence of any commercial or financial relationships that could be construed as a potential conflict of interest.
